# One step generation of customizable gRNA vectors for multiplex CRISPR approaches through string assembly gRNA cloning (STAgR)

**DOI:** 10.1371/journal.pone.0196015

**Published:** 2018-04-27

**Authors:** Christopher T. Breunig, Tamara Durovic, Andrea M. Neuner, Valentin Baumann, Maximilian F. Wiesbeck, Anna Köferle, Magdalena Götz, Jovica Ninkovic, Stefan H. Stricker

**Affiliations:** 1 MCN Junior Research Group, Munich Center for Neurosciences, Ludwig-Maximilian-Universität, BioMedical Center, Planegg-Martinsried, Germany; 2 Epigenetic Engineering, Institute of Stem Cell Research, Helmholtz Zentrum, German Research Center for Environmental Health, Planegg-Martinsried, Germany; 3 Graduate School of Systemic Neurosciences, Ludwig-Maximilians-University, Munich, Germany; 4 Neurogenesis and Regeneration, Institute of Stem Cell Research, Helmholtz Zentrum, German Research Center for Environmental Health, Neuherberg, Germany; 5 Neural stem cells, Institute of Stem Cell Research, Helmholtz Zentrum, German Research Center for Environmental Health, Neuherberg, Germany; 6 Physiological Genomics, BioMedical Center, Ludwig-Maximilian-Universität, Planegg-Martinsried, Germany; Justus Liebig Universitat Giessen, GERMANY

## Abstract

Novel applications based on the bacterial CRISPR system make genetic, genomic, transcriptional and epigenomic engineering widely accessible for the first time. A significant advantage of CRISPR over previous methods is its tremendous adaptability due to its bipartite nature. Cas9 or its engineered variants define the molecular effect, while short gRNAs determine the targeting sites. A majority of CRISPR approaches depend on the simultaneous delivery of multiple gRNAs into single cells, either as an essential precondition, to increase responsive cell populations or to enhance phenotypic outcomes. Despite these requirements, methods allowing the efficient generation and delivery of multiple gRNA expression units into single cells are still sparse. Here we present STAgR (String assembly gRNA cloning), a single step gRNA multiplexing system, that obtains its advantages by employing the N20 targeting sequences as necessary homologies for Gibson assembly. We show that STAgR allows reliable and cost-effective generation of vectors with high numbers of gRNAs enabling multiplexed CRISPR approaches. Moreover, STAgR is easily customizable, as vector backbones as well as gRNA structures, numbers and promoters can be freely chosen and combined. Finally, we demonstrate STAgR’s widespread functionality, its efficiency in multi-targeting approaches, using it for both, genome and transcriptome editing, as well as applying it in vitro and in vivo.

## Introduction

The adaptation of CRISPR as a molecular tool has been the most recent revolution in synthetic biology [1], since several groups have transformed components of this prokaryotic immune system to acquire programmable genomic targeting [2–4]. The nuclease Cas9, the only protein component in CRISPR, has the extraordinary feature of finding and binding those sequences in the genome, that are encoded in a small RNA, the guide or gRNA. While originally developed to induce double strand brakes on single sites [4], several refinements and modifications of both the protein as well as the RNA part allow at present a large spectrum of experimental strategies ranging from epigenome engineering to transcriptional activation/ repression [5, 6]. Many of these are, however, strictly dependent on the simultaneous delivery and expression of multiple gRNAs. This includes, for example, the use of Cas9 nickases [7], the induction of translocations [8, 9], medium scale deletions [10], larger genomic alterations [11, 12], CRISPR mediated generation of conditional alleles [10, 13], generation of concomitant mutations [14] and long term lineage tracing using CRISPR [15]. Furthermore, a large number of CRISPR strategies rely on multiple targeting sites, sometimes in proximity to each other, to obtain maximal effect sizes; for example when fusion proteins of the enzymatically dead dCas9 with transcriptional activators [16–18] or chromatin enzymes [19] are used for transcriptional engineering, epigenome editing [20] or cellular reprogramming [16, 21]. However, combining multiple individual gRNA expression vectors to achieve expression of multiple gRNAs in single cells has its limits, in vitro, as well as in vivo, as the fraction of cells expressing a complete set of gRNAs is decreasing with the number of gRNAs used, and those that do, rarely receive stoichiometric levels. Besides these approaches, which are strictly depending on the availability of multiple gRNAs in single cells, there is also a more general need for quick and cost-effective vector generation. Due to differences in targeting efficiencies and chromatin topology, testing a significant number of potential gRNA sequences is advisable for most experimental setups. Thus, the availability of customization strategies would constitute decisive advantages. Here we report a simple and cost-effective one step method to generate functional expression vectors for multiple gRNA delivery with high reliability and a large number of options for customization.

## Materials and methods

A detailed protocol for STAgR cloning is available supplementary (S1 File). HeLa cells (gift from Stephan Beck) were cultured in DMEM medium (Dulbecco’s modified eagle medium) supplemented with 10% (v/v) FCS and 1% penicillin/streptomycin (10000 U/ml penicillin, 10000 μg/ml streptomycin). Cells were grown in a monolayer in cell culture dishes at 37°C and 5% CO2. P19 (obtained from ATCC) were cultured in identical conditions, but supplemented with 1% NEAAs (Gibco). For each transfection 250,000 cells/well were seeded into 6-well plates. 2 μg of each STAgR and control plasmid was transfected per sample using Lipofecatmin2000 according to the manufacturer’s instructions. Seven days after the transfection cells were analysed by flow cytometry or qPCR respectively. For flow analysis, cells have been trypsinized, washed once with PBS and directly analyzed on a FACSAriaIII^™^(Becton Dickinson) flow cytometer. For each sample, GFP signal of 10000 cells has been recorded. RNA was extracted from P19 cells using the Quiagen RNAeasy Mini Kit according the manufacturer’s instructions. 100 ng RNA was reverse transcribed using the Thermo Fisher cDNA first strand kit. qPCR reactions were performed on an Applied Biosystems^™^ QuantStudio^™^ 6 Flex Real-Time PCR System. Each 10-μl reaction consisted of 5 μl of cDNA, 5μl PowerUp^™^ SYBR^™^ Green Master Mix (Thermo Scientific), and appropriate amount of primers. The amount of the target transcript was quantified relative to Gapdh as a reference. Each sample was assayed at least in triplicates.

For *in vivo* experiments STAgR plasmids and a wildtype Cas9 expression construct were mixed with Fast Green dye (0.3 mg ml^−1^; Sigma). 400ng (STAgR plasmids) and 800ng (Expression plasmid containing a Cas9 expression cassette derived from [22]) in 1 μl artificial CSF solution have been injected per fish representing a molar plasmid ratio of approximately 1:1. To conduct the injections fish were anaesthetized in 0.02% MS222 and immobilized in a sponge. A small hole was generated in the skull using a micro-knife to expose the brain tissue between the telencephalon and the optic tectum (Fine Science Tools). Subsequently, ventricular injections of the plasmid DNA were performed as previously described [23] using a glass capillary (Harvard Apparatus) and a pressure injector (200hPa, Femtojet^®^, Eppendorf). Five electrical pulses (amplitude: 65V; duration: 25ms; intervals: 1s) were delivered with a square-wave pulse generator TSS20 Ovodyne (Intracel) or by using the ECM830 square wave electroporator (BTX Harvard Apparatus). After the electroporation, fish were awakened in aerated water and kept in their normal husbandry conditions for 7 days until sacrifice and immunohistochemical analysis. All included animal work (and protocols) have been approved by the Goverment of Upper Bavaria (AZ 55.2-1-54-2532-09-16). For anesthesia, 0.02% MS222 and for euthanasia, an MS222 overdose (as approved in the above mentioned protocol) was used.

## Results and discussion

**Fig 1:**
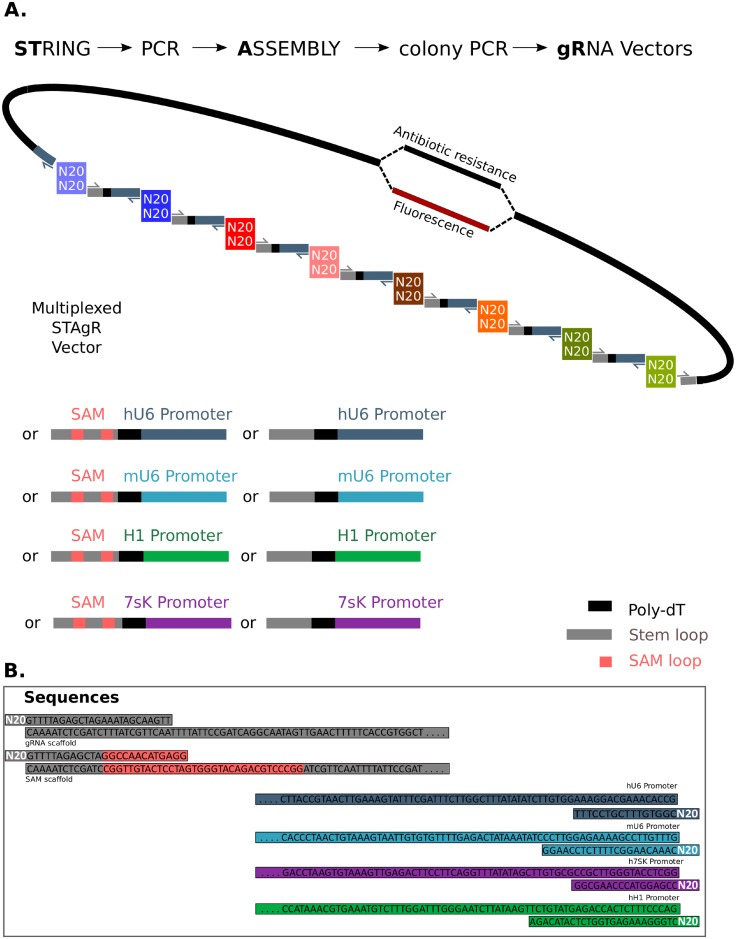
The STAgR protocol. (A) An Overview over STAgR procedure. STAgR allows simple and fast generation of multiplexing vectors in one overnight reaction. STAgR is also highly customizable as diverse strings and vectors can be used to assemble expression cassettes with different promoters and gRNA scaffolds. (B) Sequences of overhang primers used for generation of STAgR vectors.

An optimal method for the routine generation of multiplex gRNA vectors would combine simplicity with speed, would be cost-effective, efficient and highly customizable. Aiming to meet these requirements we designed a cloning strategy (STAgR) based on enzymatic assembly of short gRNA expression units, amplified from a single DNA fragment as a template, the String (S1 Fig). To avoid the need to purchase new strings for each individual gRNA, we omitted the N20 targeting sequence on the DNA fragment, containing only the gRNA hairpin, a transcriptional stop signal (poly dT) and a human U6 promoter. Instead, we provide the individual N20 sequences by short overhang primers used to amplify the String by PCR (Fig 1). In contrast to existing cloning strategies STAgR provides three distinct advantages: (1) any chosen gRNA expression unit can be amplified from the same DNA String; (2) any specific number or chosen combination of gRNA transcription units can be cloned using one single String, since the N20 targeting sequences provides the necessary homology for Gibson assembly [24]; (3) substitution and combination of different Strings as PCR template allows straight-forward customization to different Cas9- or expression-systems.

**Fig 2:**
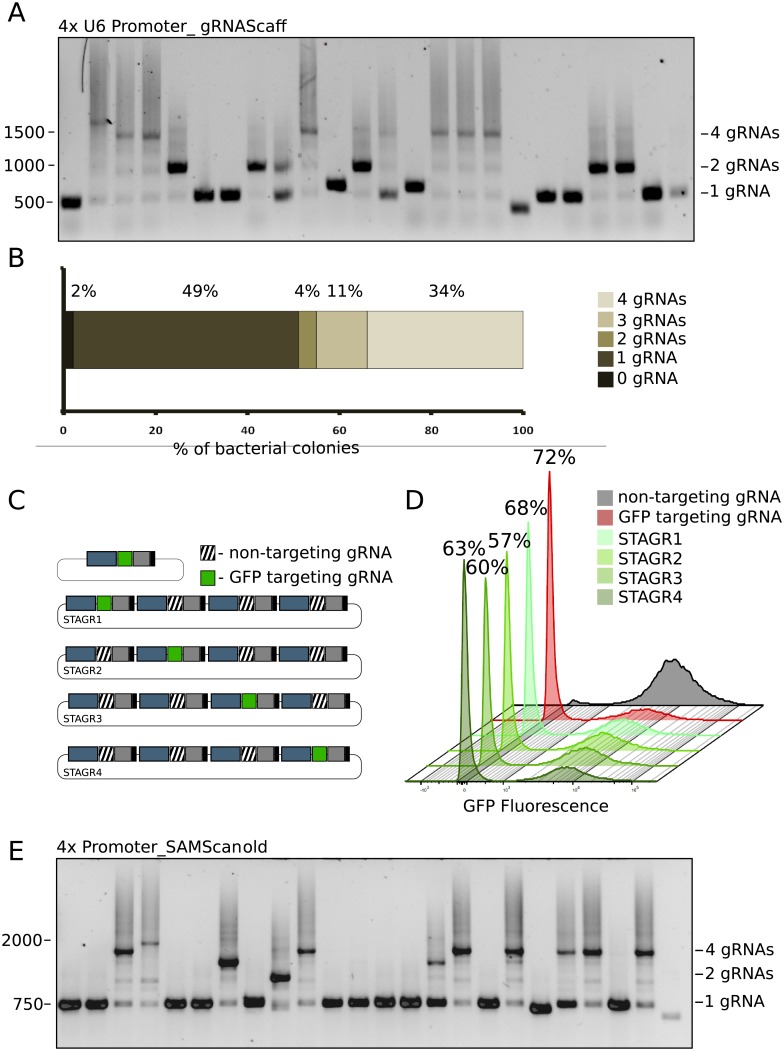
Functional validation of STAgR. (A) Colony PCR of a 4xSTAgR reaction (using a string sequence containing a hU6 promoter and a canonical gRNA scaffold). 24 bacterial colonies are shown, of which six present the amplicon size indicative of the full length reaction (1596 bp). Additionally marked are amplicon sizes indicative of two (823 bp) and single gRNAs (458 bp). (B) Quantification of cloning efficiencies from three different 4xSTAgR reactions (n = 130). (C) A schematic showing constructs used for functional validation of STAgR gRNAs. A gRNA targeting the GFP ORF was either delivered in a single gRNA expression vector or on each of four different positions in STAgR vectors. (D) Functional validation of STAgR vectors shown in Fig 2C. HeLa cells stably expressing d2GFP and Cas9 have been transfected with vectors depicted above. Flow cytometry indicates that STAgR constructs are similarly efficient in mutating the ORF of GFP compared to a single gRNA vector. (E) Colony PCR of a 4xSTAgR reaction using four different promoters and SAM loop scaffolds. 24 bacterial colonies are shown, of which seven colonies incorporated the amplicon size indicative of the full length reaction (2043 bp). Shorter amplicons are indicative of gRNA subsets, which vary in size, depending on the incorporated promoter.

First, we tested the feasibility of STAgR by aiming to clone three different sets of four individual gRNAs into a standard gRNA expression vector, pgRNA1 [25, 26]. Following a standard PCR and Gibson assembly protocol (S1 File, S1 Fig), we routinely achieved hundreds of bacterial Ampicillin resistant colonies, indicating a large number of transformation events. To avoid unnecessary time- and cost-consuming vector preparation and sequencing, we devised a robust PCR strategy to quickly characterize the accuracy of the transformed vectors in individual bacterial colonies (S1 File, Fig 2A). This enabled us not only to concentrate downstream analysis on a distinct set of vectors, but also to comprehensively characterize STAgR efficiencies. The quantification presented in Fig 2B and the example of an analytical gel in Fig 2A shows that the tested STAgR strategy is successful in generating individual multiplexed gRNA vectors. Indeed, over a third (34%, (n = 130)) of bacterial clones possessed the envisaged four gRNA expression units, which, as confirmed by subsequent Sanger sequencing, have been matching the in silico designed gRNA vector perfectly in most tested cases (90%, 5 clones each sequenced in 8 independent experiments; n = 40). This indicated that likely a small number of bacterial clones are sufficient to routinely retrieve immediately at least one impeccable plasmid.

Vectors containing multiple gRNA expression cassettes in close proximity are crucially dependent on efficient promoter and terminator sequences as transcriptional interference and/or transcriptional run-through hamper the functionality of individual gRNA sequences. Since it is still a complicated task to characterize these undesirable effects, as well as to determine the integrity and expression of individual gRNAs directly, we instead employed instead a genetic assay to quantify whether the effectiveness of a gRNA cassette changes with its position on an otherwise identical STAgR plasmid. We incorporated a single GFP-targeting gRNA into four STAgR plasmids and compared them directly to standard vectors containing only one single gRNA expression unit. We chose the assembly in such a way, that the GFP-targeting gRNA becomes incorporated on four different positions between three non-GFP-targeting control gRNA units (Fig 2C and 2D). We transfected human HeLa cells, stably expressing Cas9 and GFP, with the above mentioned gRNA expression plasmids and found that when using a single gRNA expression vector 72% of cells lost GFP expression after eight days, indicating the creation of detrimental ORF mutations. Reassuringly, STAgR plasmids containing the same GFP targeting gRNA on any of the four positions (next to non-GFP-targeting control gRNAs) triggered comparable GFP loss in site-by-site experiments, indicating similar functionality to single gRNA expression plasmids and suggesting absence of transcriptional interference or read-through in the STAgR vectors.

To demonstrate the customizability of the presented STAgR protocol, we generated a series of Strings to be used as alternative templates (Fig 1). We tested not only the adaptability of STAgR to different promoters, but also structurally different gRNA sequences. Shown as an example in Fig 2E is the assembly of four gRNAs, driven by four different promoter (human U6, mouse U6, 7SK and H1) and each containing additional RNA structures (one or two SAM loops) 3' from the gRNA hairpin to allow the targeting of MS2-fusion proteins to chosen genomic sites in addition to Cas9 fusion proteins [27]. One step STAgR assembly of this highly customized and combinatorial strategy proofed to be of similar efficiency as the STAgR strategy described before (ca. 30%, Fig 2E) and indicates a decisive advantage for the use of this method when combining different Cas9 variants, MS2 fusion proteins or dCas9 effectors

**Fig 3:**
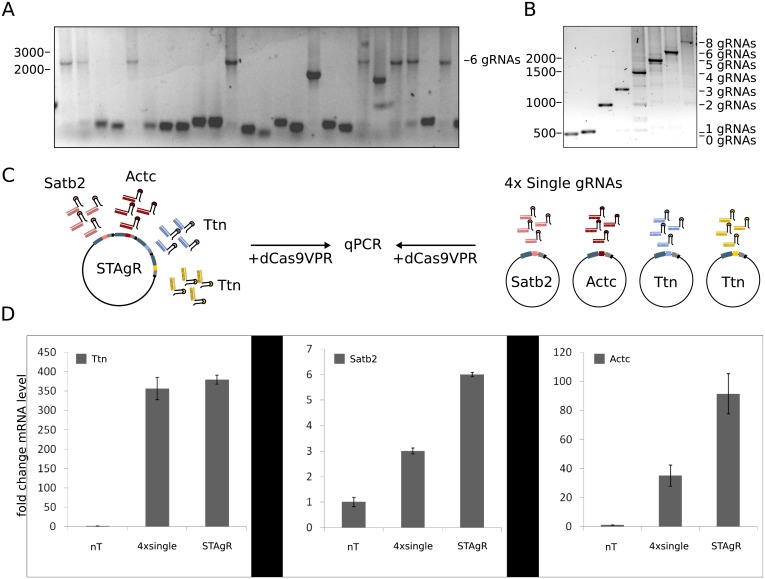
Application of STAgR. (A) Colony PCR of a 6xSTAgR reaction using two different promoters as well as both, the canonical and the SAM loop gRNA scaffold. The gel shows a colony PCR of 22 bacterial colonies, of which seven showed the amplicon indicative of the full length STAgR reaction (2444bp). (B) Exemplary colony PCR of STAgR constructs with 0 to 8 gRNA expression cassettes. (C) A STAgR plasmid containing four gRNAs or a mixture of four single gRNA plasmids have been transfected into P19 Cells expressing dCas9-VPR. (D) After 7 days mRNA was extracted and transcript levels of target genes have been compared via qPCR. Error bars depict standard errors of the mean.

To further investigate the limits of the STAgR protocol we increased the number of gRNA cassettes to be incorporated in one single reaction step. As depicted in Fig 3A, increasing the number of individual gRNA cassettes to six did not change the efficiency of the STAgR approach. A significant proportion of bacterial colonies were indicative of full gRNA incorporation in the PCR assay (30%, n = 24) and all those tested were revealing perfect assembly when sequenced (S2A Fig). Following the STAgR protocol we routinely generate vectors with up to eight expression units, the maximum number of units we have tested so far (Fig 3B) and to our knowledge amongst the highest number of gRNA units generated on single CRISPR vectors by any method. Moreover, colony PCRs also reveal, that those STAgR clones, which apparently do not contain all gRNA expression units in completion, possess mostly insert sizes representing integer multiples of single gRNA expression units, something we have observed in each experiment we have conducted so far (Figs 2A, 2E and 3A). Thus we employed Sanger sequencing to investigate, the origin of the assembly failure and the sequences of the resulting plasmids. Interestingly, nearly all (87%, n = 15) of those truncated plasmids contained in silico designed sequences without a cloning scar, breakage points or sequence repetition, but were entirely lacking one or more gRNA expression units (for examples see S2B–S2E Fig). This surprising result is due to a low-probability alignment of String sequences during assembly and offers the unexpected possibility to use STAgR not only for the generation of multiplex gRNA vectors, but also for the simultaneous obtainment of gRNA subsets.

To demonstrate the added value of combining multiple gRNAs on single vectors, we produced a STAgR plasmid containing four gRNAs, of which one is targeting the promoter of the neuronal gene Satb2, one is targeting the promoter of the cardiac muscle actin gene Actc1 and the last two are binding the promoter region of the gene Ttn1 (Fig 3C). dCas9-VPR expressing P19 cells transfected with this STAgR plasmid upregulate two of three of these genes substantially stronger than cells receiving only a mixture of single gRNAs, indicating a distinct advantage for the use of STAgR in transcript activation over conventional approaches (Fig 3D).

**Fig 4:**
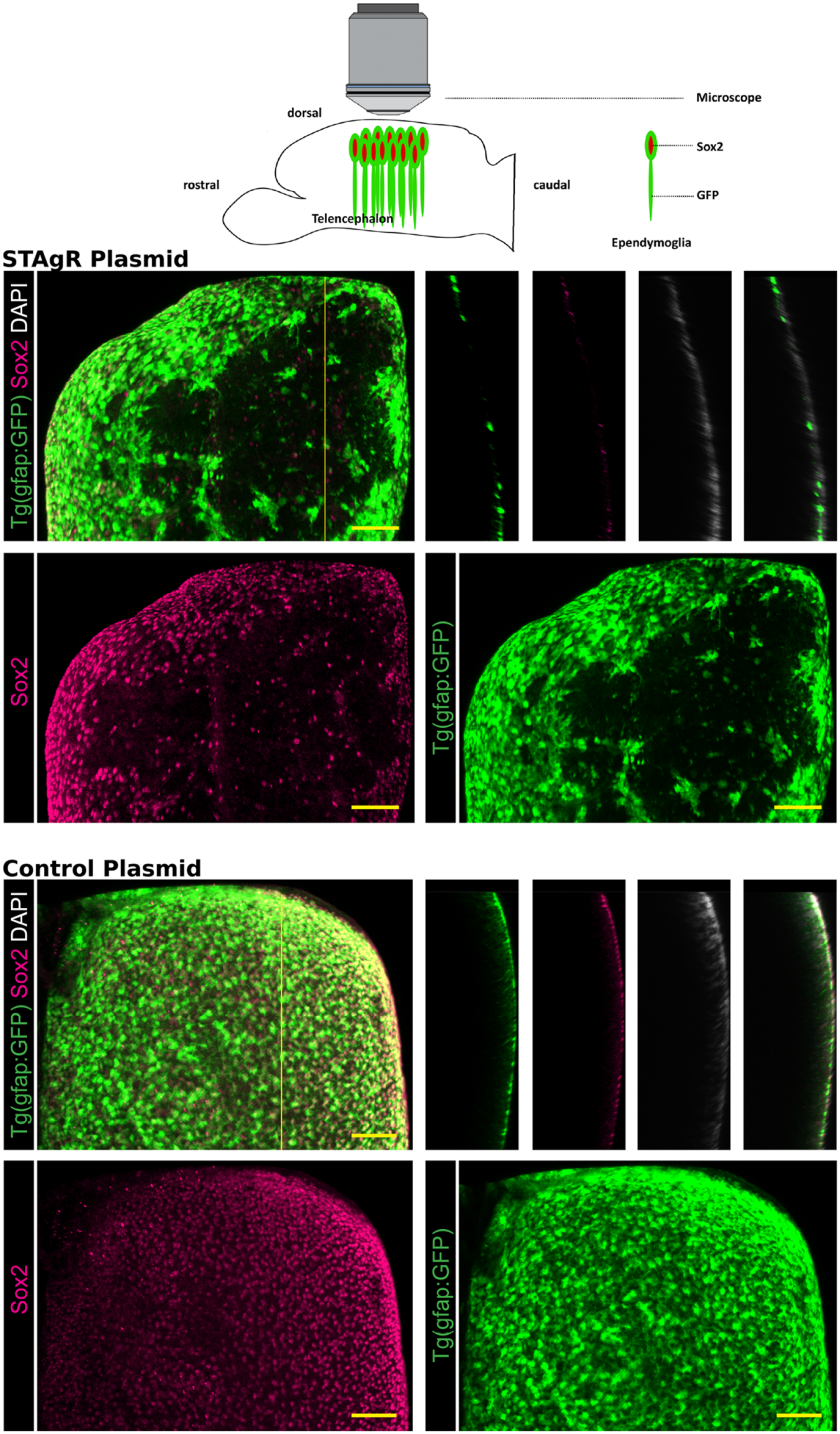
In vivo application of STAgR. STAgR constructs allow simultaneous genetic deletions in vivo. Above: Imaging setup from whole mount adult zebrafish brains. Below: 3D reconstructions of whole mount Tg(gfap:GFP) zebrafish telencephali (depicted from above). GFP+ and Sox2+ ependymoglia have been electroporated with STAgR targeting GFP and Sox2 (above) or a vector control (below) together with a Cas9 expression vector. Scale bar represents 50μm.

To test whether the in vivo use of STAgR plasmids allows efficient simultaneous disruption of multiple genes in individual cells, we employed expression vectors commonly used in zebrafish and used the protocol at hand to incorporate three gRNAs targeting GFP and Sox2 or a control STAgR vector. Subsequently, we electroporated ependymoglia in the brains of three and a half months old GFAP-GFP transgenic zebrafish, Tg(gfap:GFP) (n = 6), as previously done [28, 29] in three independent experiments using STAgR plasmids and a wildtype Cas9 expression construct (Fig 4 [22]). Seven days later we analyzed the outcome, which proved to be highly reproducible over animals and experiments. As depicted in Fig 4, a large number of ependymoglia lost GFP and Sox2 expression, while in the control electroporated brains ependymoglia cells continuously express both proteins. To our knowledge this represents the first CRISPR mediated gene knockout in adult fish brains. Moreover, most cells negative for one protein were also devoid of the other, certifying an efficient accomplishment of multiple gene targeting using STAgR in vivo.

## Conclusions

Efficient multi-gene and–locus targeting provides a critical bottleneck for the implementation of the ever increasing toolbox of CRISPR based methods. Combinations of a large number of gRNA expression vectors have the disadvantage that only a subset of cells receives all gRNA sequences. Furthermore, the limited number of available antibiotic and fluorescent selection markers makes this subset not even accessible for selective analysis. The synthesis of multi-gRNA expression vectors is slow and expensive and thus impedes the comprehensive validation of different targeting efficiencies and gRNA sequences, as well as a straight forward customization of vector backbones and CRISPR systems for most experimental setups. In contrast to this, STAgR is fast, cheap and highly efficient. Employing the protospacer sequences of the gRNAs as sources of homology for Gibson assembly enables simple and customizable vector generation for multi-gene and multi-locus targeting. Following the attached protocol, vectors allowing the simultaneous targeting of high numbers of genes or loci can be generated efficiently in one over-night reaction. Moreover, the STAgR protocol does not depend on expensive or restricted materials or skill sets; its simplicity makes the application universally available (see detailed supplementary manual, S1 File). Only few strategies have been published so far which allow the multiplexed generation of multiple gRNA vectors [30–34]. These methods have much practical value, but they either lack the simplicity of vector generation in a single step, the high number of individual gRNA expression cassettes that can be combined, the customizability and/or the independence from purchasing new large DNA fragments for each individual gRNA sequence. STAgR, a one-step method for the generation of functional gRNA vectors, is reliable, highly customizable, simple and efficient, to prove its effectiveness we used it to generate the first in vivo gene targeting in the adult fish brain.

### Accession numbers

Plasmids for STAgR cloning can be obtained from Addgene and sequences from figshare (https://figshare.com/projects/One_step_generation_of_customizable_gRNA_vectors_for_multiplex_CRISPR_approaches_through_string_assembly_gRNA_cloning_STAgR_/31046). The ID numbers are pcDNA_STAGR_SAMScaffold_hH1 (ID 102847), pcDNA_STAGR_SAMScaffold_h7SK (ID 102846), pcDNA_STAGR_SAMScaffold_mU6 (ID 102845), pcDNA_STAGR_gRNAScaffold_mU6 (ID 102844), pcDNA_STAGR_SAMScaffold_hU6 (ID 102843), pcDNA_STAGR_gRNAScaffold_h7SK (ID 102842), pcDNA_STAGR_gRNAScaffold_hH1 (ID 102841), pcDNA_STAGR_gRNAScaffold_hU6 (ID 102840), STAgR_mCherry (ID 102993), STAgR_Neo (ID 102992).

## Supporting information

S1 Fig Overview of the STAgR protocol. (EPS)

S2 Fig Sanger sequencing of five clones acquired in one single 6xSTAgR reaction. Upper left: scheme depicting complete and partial incorporations. Upper right: colony PCRs of five random clones sequenced. Shown below are sequences obtained demonstrating accurate incorporation of 6 (A), 4 (B), 3 (C), 2 (D) or 1 (E) gRNA cassettes.
(TIFF)

S1 File A detailed protocol for STAgR cloning. (PDF)

## References

[1] Ledford H. CRISPR: gene editing is just the beginning. Nature. 2016;531(7593):156–9. doi: 10.1038/531156a . 26961639 10.1038/531156a

[2] Cong L, Ran FA, Cox D, Lin S, Barretto R, Habib N, et al Multiplex genome engineering using CRISPR/Cas systems. Science. 2013;339(6121):819–23. doi: 10.1126/science.1231143 23287718 10.1126/science.1231143 PMC3795411

[3] Jinek M, East A, Cheng A, Lin S, Ma E, Doudna J. RNA-programmed genome editing in human cells. eLife. 2013;2:e00471 doi: 10.7554/eLife.00471 23386978 10.7554/eLife.00471 PMC3557905

[4] Jinek M, Chylinski K, Fonfara I, Hauer M, Doudna JA, Charpentier E. A programmable dual-RNA-guided DNA endonuclease in adaptive bacterial immunity. Science. 2012;337(6096):816–21. doi: 10.1126/science.1225829 . 22745249 10.1126/science.1225829 PMC6286148

[5] Köferle A, Stricker SH, Beck S. Brave new epigenomes: the dawn of epigenetic engineering. Genome Med. 2015;7(1):59 doi: 10.1186/s13073-015-0185-8 26089986 10.1186/s13073-015-0185-8 PMC4472160

[6] Chavez A, Scheiman J, Vora S, Pruitt BW, Tuttle M, E PRI, et al Highly efficient Cas9-mediated transcriptional programming. Nat Methods. 2015;12(4):326–8. doi: 10.1038/nmeth.3312 25730490 10.1038/nmeth.3312 PMC4393883

[7] Shen B, Zhang W, Zhang J, Zhou J, Wang J, Chen L, et al Efficient genome modification by CRISPR-Cas9 nickase with minimal off-target effects. Nat Methods. 2014;11(4):399–402. doi: 10.1038/nmeth.2857 . 24584192 10.1038/nmeth.2857

[8] Lekomtsev S, Aligianni S, Lapao A, Burckstummer T. Efficient generation and reversion of chromosomal translocations using CRISPR/Cas technology. BMC Genomics. 2016;17(1):739 doi: 10.1186/s12864-016-3084-5 27640184 10.1186/s12864-016-3084-5 PMC5027121

[9] Jiang J, Zhang L, Zhou X, Chen X, Huang G, Li F, et al Induction of site-specific chromosomal translocations in embryonic stem cells by CRISPR/Cas9. Scientific reports. 2016;6:21918 doi: 10.1038/srep21918 26898344 10.1038/srep21918 PMC4761995

[10] Yang H, Wang H, Shivalila CS, Cheng AW, Shi L, Jaenisch R. One-step generation of mice carrying reporter and conditional alleles by CRISPR/Cas-mediated genome engineering. Cell. 2013;154(6):1370–9. doi: 10.1016/j.cell.2013.08.022 23992847 10.1016/j.cell.2013.08.022 PMC3961003

[11] Wang L, Shao Y, Guan Y, Li L, Wu L, Chen F, et al Large genomic fragment deletion and functional gene cassette knock-in via Cas9 protein mediated genome editing in one-cell rodent embryos. Scientific reports. 2015;5:17517 doi: 10.1038/srep17517 26620761 10.1038/srep17517 PMC4664917

[12] Zhang L, Jia R, Palange NJ, Satheka AC, Togo J, An Y, et al Large genomic fragment deletions and insertions in mouse using CRISPR/Cas9. PLoS One. 2015;10(3):e0120396 doi: 10.1371/journal.pone.0120396 25803037 10.1371/journal.pone.0120396 PMC4372442

[13] Yang H, Wang H, Jaenisch R. Generating genetically modified mice using CRISPR/Cas-mediated genome engineering. Nat Protoc. 2014;9(8):1956–68. doi: 10.1038/nprot.2014.134 . 25058643 10.1038/nprot.2014.134

[14] Wang H, Yang H, Shivalila CS, Dawlaty MM, Cheng AW, Zhang F, et al One-step generation of mice carrying mutations in multiple genes by CRISPR/Cas-mediated genome engineering. Cell. 2013;153(4):910–8. doi: 10.1016/j.cell.2013.04.025 23643243 10.1016/j.cell.2013.04.025 PMC3969854

[15] Kalhor R, Mali P, Church GM. Rapidly evolving homing CRISPR barcodes. Nat Methods. 2017;14(2):195–200. doi: 10.1038/nmeth.4108 27918539 10.1038/nmeth.4108 PMC5322472

[16] Balboa D, Weltner J, Eurola S, Trokovic R, Wartiovaara K, Otonkoski T. Conditionally Stabilized dCas9 Activator for Controlling Gene Expression in Human Cell Reprogramming and Differentiation. Stem Cell Reports. 2015;5(3):448–59. doi: 10.1016/j.stemcr.2015.08.001 26352799 10.1016/j.stemcr.2015.08.001 PMC4618656

[17] Chakraborty S, Ji H, Kabadi AM, Gersbach CA, Christoforou N, Leong KW. A CRISPR/Cas9-based system for reprogramming cell lineage specification. Stem Cell Reports. 2014;3(6):940–7. doi: 10.1016/j.stemcr.2014.09.013 25448066 10.1016/j.stemcr.2014.09.013 PMC4264059

[18] Maeder ML, Linder SJ, Cascio VM, Fu Y, Ho QH, Joung JK. CRISPR RNA-guided activation of endogenous human genes. Nat Methods. 2013;10(10):977–9. doi: 10.1038/nmeth.2598 23892898 10.1038/nmeth.2598 PMC3794058

[19] Hilton IB, D'Ippolito AM, Vockley CM, Thakore PI, Crawford GE, Reddy TE, et al Epigenome editing by a CRISPR-Cas9-based acetyltransferase activates genes from promoters and enhancers. Nat Biotechnol. 2015 doi: 10.1038/nbt.3199 . 25849900 10.1038/nbt.3199 PMC4430400

[20] Stricker SH, Koferle A, Beck S. From profiles to function in epigenomics. Nat Rev Genet. 2017;18(1):51–66. doi: 10.1038/nrg.2016.138 . 27867193 10.1038/nrg.2016.138

[21] Black JB, Adler AF, Wang HG, D'Ippolito AM, Hutchinson HA, Reddy TE, et al Targeted Epigenetic Remodeling of Endogenous Loci by CRISPR/Cas9-Based Transcriptional Activators Directly Converts Fibroblasts to Neuronal Cells. Cell Stem Cell. 2016;19(3):406–14. doi: 10.1016/j.stem.2016.07.001 27524438 10.1016/j.stem.2016.07.001 PMC5010447

[22] Ran FA, Hsu PD, Wright J, Agarwala V, Scott DA, Zhang F. Genome engineering using the CRISPR-Cas9 system. Nat Protoc. 2013;8(11):2281–308. doi: 10.1038/nprot.2013.143 24157548 10.1038/nprot.2013.143 PMC3969860

[23] Kizil C, Brand M. Cerebroventricular microinjection (CVMI) into adult zebrafish brain is an efficient misexpression method for forebrain ventricular cells. PLoS One. 2011;6(11):e27395 doi: 10.1371/journal.pone.0027395 22076157 10.1371/journal.pone.0027395 PMC3208640

[24] Gibson DG, Young L, Chuang RY, Venter JC, Hutchison CA 3rd, Smith HO. Enzymatic assembly of DNA molecules up to several hundred kilobases. Nat Methods. 2009;6(5):343–5. doi: 10.1038/nmeth.1318 . 19363495 10.1038/nmeth.1318

[25] Koferle A, Worf K, Breunig C, Baumann V, Herrero J, Wiesbeck M, et al CORALINA: a universal method for the generation of gRNA libraries for CRISPR-based screening. BMC Genomics. 2016;17(1):917 doi: 10.1186/s12864-016-3268-z 27842490 10.1186/s12864-016-3268-z PMC5109649

[26] Koferle A, Stricker SH. A universal protocol for large-scale gRNA library production from any DNA source. Journal of Visualized Experiments. in press. 10.3791/56264 PMC5755530 29286403

[27] Konermann S, Brigham MD, Trevino AE, Joung J, Abudayyeh OO, Barcena C, et al Genome-scale transcriptional activation by an engineered CRISPR-Cas9 complex. Nature. 2014 doi: 10.1038/nature14136 . 25494202 10.1038/nature14136 PMC4420636

[28] Barbosa JS, Sanchez-Gonzalez R, Di Giaimo R, Baumgart EV, Theis FJ, Gotz M, et al Neurodevelopment. Live imaging of adult neural stem cell behavior in the intact and injured zebrafish brain. Science. 2015;348(6236):789–93. doi: 10.1126/science.aaa2729 . 25977550 10.1126/science.aaa2729

[29] Barbosa JS, Di Giaimo R, Gotz M, Ninkovic J. Single-cell in vivo imaging of adult neural stem cells in the zebrafish telencephalon. Nat Protocols. 2016;11(8):1360–70. doi: 10.1038/nprot.2016.077 27362338 10.1038/nprot.2016.077

[30] Xie K, Minkenberg B, Yang Y. Boosting CRISPR/Cas9 multiplex editing capability with the endogenous tRNA-processing system. Proc Natl Acad Sci U S A. 2015;112(11):3570–5. doi: 10.1073/pnas.1420294112 25733849 10.1073/pnas.1420294112 PMC4371917

[31] Kabadi AM, Ousterout DG, Hilton IB, Gersbach CA. Multiplex CRISPR/Cas9-based genome engineering from a single lentiviral vector. Nucleic Acids Res. 2014;42(19):e147 doi: 10.1093/nar/gku749 25122746 10.1093/nar/gku749 PMC4231726

[32] Sakuma T, Nishikawa A, Kume S, Chayama K, Yamamoto T. Multiplex genome engineering in human cells using all-in-one CRISPR/Cas9 vector system. Scientific reports. 2014;4:5400 doi: 10.1038/srep05400 24954249 10.1038/srep05400 PMC4066266

[33] Port F, Bullock SL. Augmenting CRISPR applications in Drosophila with tRNA-flanked sgRNAs. Nat Methods. 2016;13(10):852–4. doi: 10.1038/nmeth.3972 27595403 10.1038/nmeth.3972 PMC5215823

[34] Ferreira R, Skrekas C, Nielsen J, David F. Multiplexed CRISPR/Cas9 Genome Editing and Gene Regulation Using Csy4 in Saccharomyces cerevisiae. ACS Synth Biol. 2017 doi: 10.1021/acssynbio.7b00259 . 29161506 10.1021/acssynbio.7b00259

