# Supplementary material for: One step generation of customizable gRNA vectors for multiplex CRISPR approaches through string assembly gRNA cloning (STAgR)
Source: PLoS One. 2018 Apr 27;13(4):e0196015. doi: 10.1371/journal.pone.0196015 (PMC5922533; doi:10.1371/journal.pone.0196015)
Supplement: S1 File — (PDF) [file pone.0196015.s003.pdf]

STAGR- String assembly gRNA cloning

This protocol is for one- step generation of gRNA plasmids with 2-8 expression cassettes.

1. **STAGR primer design:**

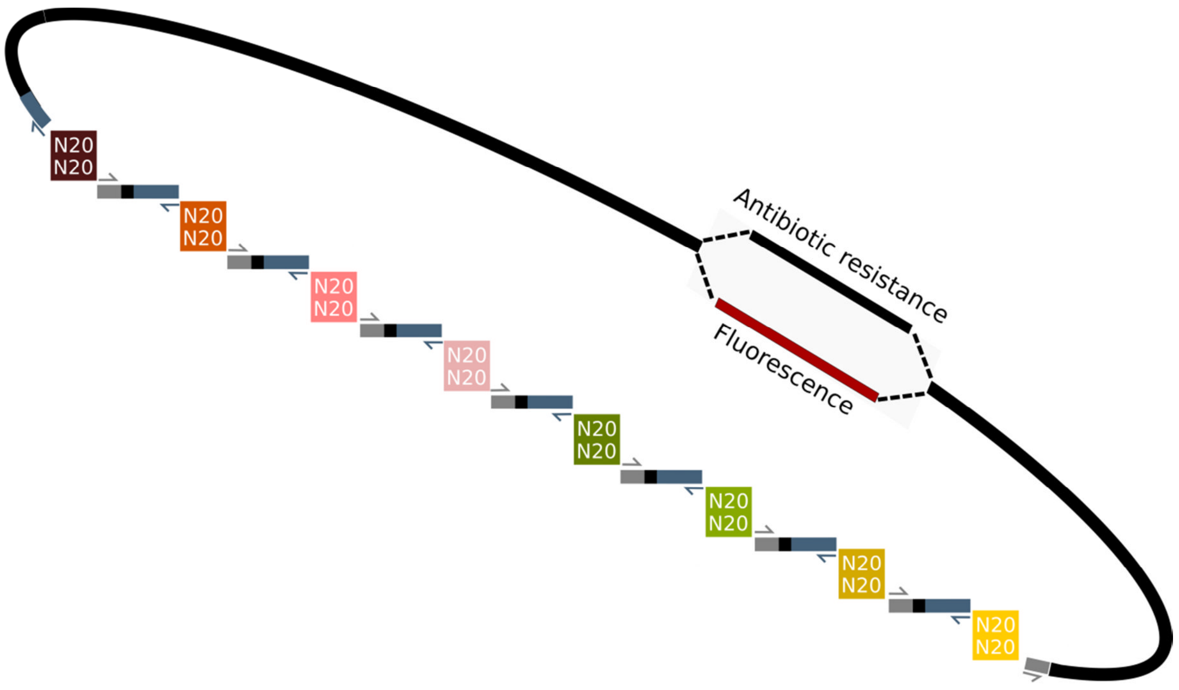

Fig.P1 Scheme of a 6xSTAgR assembly

1. Decide how many gRNA cassettes should be included in one vector, and which promoters and gRNA scaffolds should be used for each of the gRNAs. Design your gRNAs by using your preferred tool (e.g. www.benchling.com).

2. The gRNA sequences are added to the primers for amplification of the STAgR DNA string as overhangs. Sense gRNA sequences are added to the forward primer (which binds to the scaffold/SAM part of the respective STAgR string). The antisense gRNA sequence is added in front of the reverse primer sequences of the specific promoters.

Tab.P1 : Primer Sequences for gRNA scaffolds and promoters.

Forward primer for String and Vector	
scaffold_fwd	GTTTTAGAGCTAGAAATAGCAAGTT
SAM_fwd	GTTTTAGAGCTAGGCCAACATGAGG
Reverse primer for String and Vector	
hU6_rev	CGGTGTTTCGTCCTTT
mU6_rev	CAAACAAGGCTTTTCTCCAAGG
hH1_rev	CTGGGAAAGAGTGGTCTCATACAGA
h7SK_rev	CCGAGGTACCCAAGCGG

The sense gRNA sequence (Fig. P1, green) of the last gRNA and the antisense of the first gRNA (Fig. S1brown) respectively are added to the PCR primers used for amplification of the STAgR vector backbone. The remainder of the primers are used for amplification from the string, thereby attaching the sense and antisense gRNAs so as to use them as overhangs for assembly in the desired order. The first string piece is amplified with a forward primer using the first gRNA (Fig. P1 brown) as an overhang and a reverse primer using the next gRNA in the sequence in the antisense direction (Fig. P1 dark red) as an overhang. The next STAgR pieces are amplified with a forward primer (using the dark red protospacer in Fig. P1) as an overhang and a reverse primer (using the light red protospacer, Fig. P1) as an overhang and so on.

2. PCR amplification

This step generates the Gibson fragments for assembly into the vector backbone. The choice of STAgR DNA string template defines the promoter and gRNA scaffold for each gRNA in the assembled vector.

Set up every string and vector reaction as follows:

10 µl Phusion HF buffer

1 µl 10mM dNTPs

0,25 µl Primer (100 µM)

10 ng DNA template

0,5 µl Phusion Polymerase

1,5 µl DMSO

➔ Add H20 to 50µl

98°C 1.30min

98°C 10sec

59°C 30sec (for gRNA Scaffold) 68°C 30sec (for SAM loop)

72°C 30sec (inserts) / 1.30 min (vector) x38

72°C 10min

4°C ∞

To incorporate the gRNA scaffold, use “STAgR_Neo” as a template for the vector PCR, if the SAM Scaffold is the desired gRNA scaffold use “pcSAM_helper”. Analyze the amplified fragments on a 1% agarose gel (Fig. P2). Digest the vector reaction with DpnI as follows:

44,5 µl PCR reaction

5 µl 10x Cutsmart Buffer

0,5 µl DpnI (10 units, NEB)

Incubate at 37°C, 30min – 1h

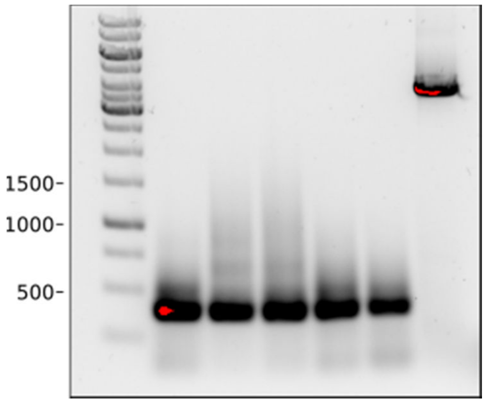

Fig. P2: STAgR PCR of different string fragment (lanes 1-5) and a circular amplification of a vector (lane 6)

1.4 Clean up STAgR pieces with AMPure XP Beads or any column-based reaction clean-up kit.

1.5 Determine DNA concentrations

3. Gibson Assembly Reaction

This step assembles the pieces previously generated by PCR into a vector with multiple gRNA expression cassettes in a single step.

All amplified gRNA expression cassettes are used in equimolar amounts.

3.1 Set up the following reactions on ice (include a no insert control):

Tab. P2 : Composition of STAgR Reaction

	2x STAgR	3-8x STAgR
Recommended DNA Ratio	vector:insert 1:1	vector:insert = 1:3
Total Amount of DNA	0.03–0.2 pmol	0.2–0.5 pmol
Gibson Assembly MasterMix (1,5x)	7,5 µl	7,5 µl
Total Volume	10 µl*	10µl*

*Total volume can be increased to 20 µl. The amount of Gibson Assembly Master Mix then has to be doubled as well.

3.2 Incubate samples in a thermocycler at 50°C for 45 to 60 minutes. Store samples on ice or at –20°C for subsequent transformation.

3.3 Directly transform half of the mix into chemically competent bacteria. Do not forget two plasmid controls, one where you directly transform the same amount of vector you put into the Gibson reaction and one actual Gibson reaction only with the vector and without any inserts.

3.4. After recovery, plate the transformed bacteria onto agar plates containing Ampicillin (100µg/ml) and incubate overnight at 37°C.

4. Colony PCR

The aim of this step is to identify bacterial colonies that harbour plasmids with the correct number of assembled gRNAs. The primers used bind outside the gRNA expression cassettes and the size of the amplicon thus corresponds to the number of gRNA expression cassettes in the vector.

StAgR_seq_fwd2: ACTGGATCCGGTACCAAGG

StAgR_seq_rev: TTACGGTTCCTGGCCTTTTG

4.1 Analyze at least 24 bacterial colonies. For each PCR reaction, pre-aliquot the appropriate number of vials with 100 µl LB medium with Ampicillin (100µg/ml).

4.2 Set up a PCR master mix (10 µl per reaction):

For 10 reactions:

10 µl Taq buffer

2µl 10mM dNTPs

0,5 µl Primer (100mM)

0,5 µl Taq Polymerase

➔ Add $H_2$0 to 100µl

4.2 Aliquot the PCR master mix into microcentrifuge tubes

4.3. Using a sterile pipette tip, pick a colony from the agar plate and insert the tip into one aliquot of the PCR master mix. Swirl the tip around gently to ensure some of the bacteria are transferred to the master mix, before transferring the tip to the corresponding aliquot of LB medium, to be cultured at 37°C for later use.

4.4. Run the PCR reactions using the following program:

PCR Programm Colony PCR:

94°C 5min

94°C 30sec

55°C 30sec

72°C 2min x38

72°C 10min

4°C ∞

Primer:

StAgR_seq_fwd2: ACTGGATCCGGTACCAAGG

StAgR_seq_rev: TTACGGTTCCTGGCCTTTTG

4.5 Analyze the PCR amplicons on a 1% agarose gel.

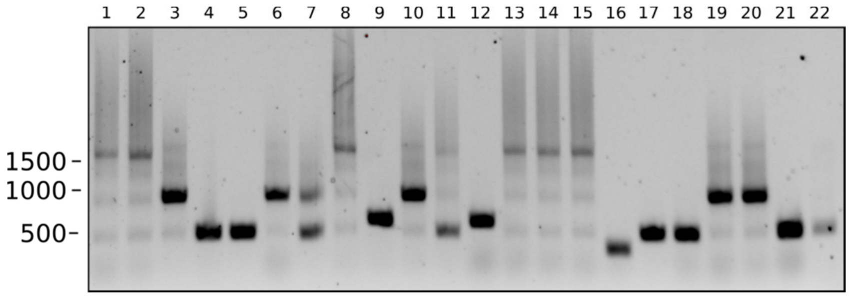

Fig. P3: Colony PCR of a 4xSTAgR reaction using hU6 promoters and canonical gRNA scaffold. 24 bacterial colonies were analyzed, of which 6 showed the estimated band of a 4x STAgR cassette (1596 bp). Vectors with two gRNA expression cassettes yield a 823 bp amplicon, a band at 458 bp corresponds to a single gRNA cassette being present in the vector.

Amplicon sizes vary according the promoter and gRNA Stem loop used.

Tab. P3 Sizes of promoters and scaffolds

hU6	265bp
hH1	225bp
mU6	316bp
H7SK	245bp
gRNA scaffold	83bp
SAMloop + gRNA scaffold	143bp

4.4 From the results of the colony PCR identify the cultures harbouring the correct vectors and inoculate a 2,5ml overnight LB culture (with 100µg/ml ampicillin). Incubate for 12h at 37°C

4.5 Extract plasmid DNA.

4.6 Sequence the plasmids using the following primers. StAgR_seq_fwd1 (GAGTTAGGGGCGGGACTATG), StAgR_seq_fwd2 (ACTGGATCCGGTACCAAGG) and StAgR_seq_rev (TTACGGTTCCTGGCCTTTTG)

Appendix

Forward primer for String and Vector:

scaffold_fwd	NNNNNNNNNNNNNNNNNNNNGTTTTAGAGCTAGAAATAGCAAGTT
SAM_fwd	NNNNNNNNNNNNNNNNNNNNGTTTTAGAGCTAGGCCAACATGAGG

Reverse primer for Sting and Vector:

hU6_rev	NNNNNNNNNNNNNNNNNNNNCGGTGTTTCGTCCTTT
mU6_rev	NNNNNNNNNNNNNNNNNNNNCAAACAAGGCTTTTCTCCAAGG
hH1_rev	NNNNNNNNNNNNNNNNNNNNCTGGGAAAGAGTGGTCTCATACAGA
h7SK_rev	NNNNNNNNNNNNNNNNNNNNCCGAGGTACCCAAGCGG

gRNA Scaffold

GTTTTAGAGCTAGAAATAGCAAGTTAAAATAAGGCTAGTCCGTTATCAACTTGAAAAAGTGGCACCGAGTCGG
TGCTTTTTTT

SAM loop

GTTTTAGAGCTAGGCCAACATGAGGATCACCCATGTCTGCAGGGCCTAGCAAGTTAAAATAAGGCTAGTCCGT
TATCAACTTGGCCAACATGAGGATCACCCATGTCTGCAGGGCCAAGTGGCACCGAGTCGGTGCTTTTTTT

h7SK

CTGCAGTATTTAGCATGCCCCACCCATCTGCAAGGCATTCTGGATAGTGTCAAAACAGCCGGAAATCAAGTCC
GTTTATCTCAAACTTTAGCATTTTGGGAATAAATGATATTTGCTATGCTGGTTAAATTAGATTTTAGTTAAATTTC
CTGCTGAAGCTCTAGTACGATAAGCAACTTGACCTAAGTGTAAAGTTGAGACTTCCTTCAGGTTTATATAGCTT
GTGCGCCGCTTGGGTACCTCGG

GGCTCCATGGGTTCGCCGCGTGTTCGATATATTTGGACTTCCTTCAGAGTTGAAATGTGAATCCAGTTCAACGA
ATAGCATGATCTCGAAGTCGTCCTTTAAATTGATTTTAGATTAAATTGGTCGTATCGTTTATAGTAAATAAGGGT
TTTACGATTTCAAACTCTATTTGCCTGAACTAAAGGCCGACAAAACTGTGATAGGTCTTACGGAACGTCTACCC
ACCCCGTACGATTTATGACGTC

hU6

AAGGTCGGGCAGGAAGAGGGCCTATTTCCCATGATTCCTTCATATTTGCATATACGATACAAGGCTGTTAGAG
AGATAATTAGAATTAATTTGACTGTAAACACAAAGATATTAGTACAAAATACGTGACGTAGAAAGTAATAATTT
CTTGGGTAGTTTGCAGTTTTAAAATTATGTTTTAAAATGGACTATCATATGCTTACCGTAACTTGAAAGTATTTC
GATTTCTTGGCTTTATATATCTTGTGGAAAGGACGAAACACCG

GCCACAAAGCAGGAAAGGTGTTCTATATATTTCGGTTCTTTAGCTTTATGAAAGTTCAATGCCATTCGTATACTA
TCAGGTAAAATTTTGTATTAAAATTTTGACGTTTGATGGGTTCTTTAATAATGAAAGATGCAGTGCATAAAACA
TGATTATAGAAACACAAATGTCAGTTTAATTAAGATTAATAGAGAGATTGTCGGAACATAGCATATACGTTTAT
ACTTCCTTAGTACCCTTTATCCGGGAGAAGGACGGGCTGGAA

hH1

GAACGCTGACGTCATCAACCCGCTCCAAGGAATCGCGGGCCCAGTGTCACTAGGCGGGAACACCCAGCGCGC
GTGCGCCCTGGCAGGAAGATGGCTGTGAGGGACAGGGGAGTGGCGCCCTGCAATATTTGCATGTCGCTATGT
GTTCTGGGAAATCACCATAAACGTGAAATGTCTTTGGATTTGGGAATCTTATAAGTTCTGTATGAGACCACTCT
TTCCCAG

GACCCTTTCTCACCAGAGTATGTCTTGAATATTCTAAGGGTTTAGGTTTCTGTAAAGTGCAAATACCACTAAAG
GGTCTTGTGTATCGCTGTACGTTTATAACGTCCCGCGGTGAGGGGACAGGGAGTGTCGGTAGAAGGACGGTC
CCGCGTGCGCGCGACCCACAAGGGCGGATCACTGTGACCCGGGCGCTAAGGAACCTCGCCCAACTACTGCAG
TCGCAAG

mU6

GATCCGACGCCGCCATCTCTAGGCCCGCGCCGGCCCCCTCGCACAGACTTGTGGGAGAAGCTCGGCTACTCCC
CTGCCCCGGTTAATTTGCATATAATATTTCCTAGTAACTATAGAGGCTTAATGTGCGATAAAAGACAGATAATC
TGTTCTTTTTAATACTAGCTACATTTTACATGATAGGCTTGGATTTCTATAAGAGATACAAATACTAAATTATTAT
TTTAAAAAACAGCACAAAAGGAAACTCACCCTAACTGTAAAGTAATTGTGTGTTTTGAGACTATAAATATCCCT
TGGAGAAAAGCCTTGTTTG

GTTTGTTCCGAAAAGAGGTTCCCTATAAATATCAGAGTTTTGTGTGTTAATGAAATGTCAATCCCACTCAAAGG
AAAACACGACAAAAAATTTTATTATTAAATCATAAACATAGAGAATATCTTTAGGTTCGGATAGTACATTTTAC
ATCGATCATAATTTTTCTTGTCTAATAGACAGAAAATAGCGTGTAATTCGGAGATATCAATGATCCTTTATAATA
TACGTTTAATTGGCCCCGTCCCCTCATCGGCTCGAAGAGGGTGTTCAGACACGCTCCCCCGGCCGCGCCCGGA
TCTCTACCGCCGCAGCCTAG

ML3636_gRNA

TTATCCGGTAACTATCGTCTTGAGTCCAACCCGGTAAGACACGACTTATCGCCACTGGCAGCAGCCACTGGTAA
CAGGATTAGCAGAGCGAGGTATGTAGGCGGTGCTACAGAGTTCTTGAAGTGGTGGCCTAACTACGGCTACAC
TAGAAGAACAGTATTTGGTATCTGCGCTCTGCTGAAGCCAGTTACCTTCGGAAAAAGAGTTGGTAGCTCTTGA
TCCGGCAAACAAACCACCGCTGGTAGCGGTGGTTTTTTTGTTTGCAAGCAGCAGATTACGCGCAGAAAAAAAG
GATCTCAAGAAGATCCTTTGATCTTTTCTACGGGGTCTGACGCTCAGTGGAACGAAAACTCACGTTAAGGGATT
TTGGTCATGAGATTATCAAAAAGGATCTTCACCTAGATCCTTTTAAATTAAAAATGAAGTTTTAAATCAATCTAA
AGTATATATGAGTAAACTTGGTCTGACAGTTACCAATGCTTAATCAGTGAGGCACCTATCTCAGCGATCTGTCT
ATTTCGTTCATCCATAGTTGCCTGACTCCCCGTCGTGTAGATAACTACGATACGGGAGGGCTTACCATCTGGCC
CCAGTGCTGCAATGATACCGCGAGACCCACGCTCACCGGCTCCAGATTTATCAGCAATAAACCAGCCAGCCGG
AAGGGCCGAGCGCAGAAGTGGTCCTGCAACTTTATCCGCCTCCATCCAGTCTATTAATTGTTGCCGGGAAGCT
AGAGTAAGTAGTTCGCCAGTTAATAGTTTGCGCAACGTTGTTGCCATTGCTACAGGCATCGTGGTGTCACGCTC
GTCGTTTGGTATGGCTTCATTCAGCTCCGGTTCCCAACGATCAAGGCGAGTTACATGATCCCCCATGTTGTGCA
AAAAAGCGGTTAGCTCCTTCGGTCCTCCGATCGTTGTCAGAAGTAAGTTGGCCGCAGTGTTATCACTCATGGTT
ATGGCAGCACTGCATAATTCTCTTACTGTCATGCCATCCGTAAGATGCTTTTCTGTGACTGGTGAGTACTCAACC
AAGTCATTCTGAGAATAGTGTATGCGGCGACCGAGTTGCTCTTGCCCGGCGTCAATACGGGATAATACCGCGC
CACATAGCAGAACTTTAAAAGTGCTCATCATTGGAAAACGTTCTTCGGGGCGAAAACTCTCAAGGATCTTACCG
CTGTTGAGATCCAGTTCGATGTAACCCACTCGTGCACCCAACTGATCTTCAGCATCTTTTACTTTCACCAGCGTT
TCTGGGTGAGCAAAAACAGGAAGGCAAAATGCCGCAAAAAAGGGAATAAGGGCGACACGGAAATGTTGAAT

ACTCATACTCTTCCTTTTTCAATATTATTGAAGCATTTATCAGGGTTATTGTCTCATGAGCGGATACATATTTGAA
TGTATTTAGAAAAATAAACAAATAGGGGTTCCGCGCACATTTCCCCGAAAAGTGCCACCTGACGTCGATAAGA
TACATTGATGAGTTTGGACAAACCACAACTAGAATGCAGTGAAAAAAATGCTTTATTTGTGAAATTTGTGATGC
TATTGCTTTATTTGTAACCATTATAAGCTGCAATAAACAAGTTGGGGTGGGCGAAGAACTCCAGCATGAGATCC
CCGCGCTGGAGGATCATCCAGCCGGCGTCCCGGAAAACGATTCCGAAGCCCAACCTTTCATAGAAGGCGGCG
GTGGAATCGAAATCTCGTGATGGCAGGTTGGGCGTCGCTTGGTCGGTCATTTCGAACCCCAGAGTCCCGCTCA
GAAGAACTCGTCAAGAAGGCGATAGAAGGCGATGCGCTGCGAATCGGGAGCGGCGATACCGTAAAGCACGA
GGAAGCGGTCAGCCCATTCGCCGCCAAGCTCTTCAGCAATATCACGGGTAGCCAACGCTATGTCCTGATAGCG
GTCCGCCACACCCAGCCGGCCACAGTCGATGAATCCAGAAAAGCGGCCATTTTCCACCATGATATTCGGCAAG
CAGGCATCGCCATGGGTCACGACGAGATCCTCGCCGTCGGGCATGCGCGCCTTGAGCCTGGCGAACAGTTCG
GCTGGCGCGAGCCCCTGATGCTCTTCGTCCAGATCATCCTGATCGACAAGACCGGCTTCCATCCGAGTACGTGC
TCGCTCGATGCGATGTTTCGCTTGGTGGTCGAATGGGCAGGTAGCCGGATCAAGCGTATGCAGCCGCCGCATT
GCATCAGCCATGATGGATACTTTCTCGGCAGGAGCAAGGTGAGATGACAGGAGATCCTGCCCCGGCACTTCGC
CCAATAGCAGCCAGTCCCTTCCCGCTTCAGTGACAACGTCGAGCACAGCTGCGCAAGGAACGCCCGTCGTGGC
CAGCCACGATAGCCGCGCTGCCTCGTCCTGCAGTTCATTCAGGGCACCGGACAGGTCGGTCTTGACAAAAAGA
ACCGGGCGCCCCTGCGCTGACAGCCGGAACACGGCGGCATCAGAGCAGCCGATTGTCTGTTGTGCCCAGTCA
TAGCCGAATAGCCTCTCCACCCAAGCGGCCGGAGAACCTGCGTGCAATCCATCTTGTTCAATCATGCGAAACG
ATCCTCATCCTGTCTCTTGATCAGATCCGAAAATGGATATACAAGCTCCCGGGAGCTTTTTGCAAAAGCCTAGG
CCTCCAAAAAAGCCTCCTCACTACTTCTGGAATAGCTCAGAGGCAGAGGCGGCCTCGGCCTCTGCATAAATAA
AAAAAATTAGTCAGCCATGGGGCGGAGAATGGGCGGAACTGGGCGGAGTTAGGGGCGGGATGGGCGGAGT
TAGGGGCGGGACTATGGTTGCTGACTAATTGAGATGCATGCTTTGCATACTTCTGCCTGCTGGGGAGCCTGGG
GACTTTCCACACCTGGTTGCTGACTAATTGAGATGCACTGTACAAAAAAGCAGGCTTTAAAGGAACCAATTCA
GTCGACTGGATCCGGTACCAAGGTCGGGCAGGAAGAGGGCCTATTTCCCATGATTCCTTCATATTTGCATATAC
GATACAAGGCTGTTAGAGAGATAATTAGAATTAATTTGACTGTAAACACAAAGATATTAGTACAAAATACGTG
ACGTAGAAAGTAATAATTTCTTGGGTAGTTTGCAGTTTTAAAATTATGTTTTAAAATGGACTATCATATGCTTAC
CGTAACTTGAAAGTATTTCGATTTCTTGGCTTTATATATCTTGTGGAAAGGACGAAACACCGGTTTTAGAGCTA
GAAATAGCAAGTTAAAATAAGGCTAGTCCGTTATCAACTTGAAAAAGTGGCACCGAGTCGGTGCTTTTTTTAA
GCTTGGGCCGCTCGAGGTACCTCTCTACATATGACATGTGAGCAAAAGGCCAGCAAAAGGCCAGGAACCGTA
AAAAGGCCGCGTTGCTGGCGTTTTTCCATAGGCTCCGCCCCCCTGACGAGCATCACAAAAATCGACGCTCAAG
TCAGAGGTGGCGAAACCCGACAGGACTATAAAGATACCAGGCGTTTCCCCCTGGAAGCTCCCTCGTGCGCTCT
CCTGTTCCGACCCTGCCGCTTACCGGATACCTGTCCGCCTTTCTCCCTTCGGGAAGCGTGGCGCTTTCTCATAGC
TCACGCTGTAGGTATCTCAGTTCGGTGTAGGTCGTTCGCTCCAAGCTGGGCTGTGTGCACGAACCCCCCGTTCA
GCCCGACCGCTGCGCC
